# Implementation of significant mental health service change: perceptions and concerns of a mental health workforce in the context of transformation

**DOI:** 10.1108/JHOM-06-2021-0205

**Published:** 2022-02-01

**Authors:** Margaret Glogowska, Melissa Stepney, Stephen Rocks, Mina Fazel

**Affiliations:** Nuffield Department of Primary Care Health Sciences, University of Oxford , Oxford, UK; Nuffield Department of Population Health, University of Oxford , Oxford, UK; Department of Psychiatry, University of Oxford , Oxford, UK

**Keywords:** Organisational innovation, Qualitative research, Health care professionals, Health services research, Child mental health services, Service change

## Abstract

**Purpose:**

As part of an evaluation of the nationally mandated Child and Adolescent Mental Health Services (CAMHS) “transformation” in one foundation NHS trust, the authors explored the experiences of mental health staff involved in the transformation.

**Design/methodology/approach:**

The authors employed a qualitative methodology and followed an ethnographic approach. This included observation of mental health staff involved in the transformation and informal interviews (80 h). The authors also undertook semi-structured interviews with key staff members ( *n* = 16). Data were analysed thematically.

**Findings:**

The findings fall into three thematic areas around the transformation, namely (1) rationale; (2) implementation; and (3) maintenance. Staff members were supportive of the rationale for the changes, but implementation was affected by perceived poor communication, resulting in experiences of unpreparedness and de-stabilisation. Staff members lacked time to set up the necessary processes, meaning that changes were not always implemented smoothly. Recruiting and retaining the right staff, a consistent challenge throughout the transformation, was crucial for maintaining the service changes.

**Originality/value:**

There is little published on the perceptions and experiences of mental health workforces around the CAMHS transformations across the UK. This paper presents the perceptions of mental health staff, whose organisation underwent significant “transformational” change. Staff demonstrated considerable resilience in the change process, but better recognition of their needs might have improved retention and satisfaction. Time for planning and training would enable staff members to better develop the processes and resources necessary in the context of significant service change. Developing ways for services to compare changes they are implementing and sharing good practice around implementation with each other are also vital.

## Background

In recent years, evidence shows that demand for Child and Adolescent Mental Health Services (CAMHS) in high-income nations is rising ( Atkins and Lakind, 2013 ; Hughes *et al.* , 2018 ; McGorry *et al.* , 2013 ). At the same time, UK reports such as *Future in Mind* identified that services are not set up to meet the needs of the most vulnerable, and recommended services should structure changes around a number of key principles, namely, (1) to promote resilience, prevention and early intervention; (2) to improve access to effective support; (3) to care for the most vulnerable; (4) to promote accountability and transparency; and (5) to develop the workforce ( Department of Health, 2015 ). In a similar way, the *Five Year Forward View For Mental Health* highlighted the need for “transformation” of services to improve accessibility, increase quality of care and improve health outcomes ( NHS England, 2016 ). Local areas were invited to submit “Transformation Plans” to NHS England to address the recommendations by redesigning the provision they offer and subsequently, across the UK, CAMHS have been undergoing transformational changes. This has resulted in many services moving away from a “tiered” model where, depending on their perceived need and complexity, a young person is assigned to a specific level of service corresponding to their perceived need, towards a more “integrated” model of service.

These changes align with the THRIVE model ( Wolpert *et al.* , 2016 ), which advocates principles of service organisation, highlighting five main areas: “Thriving”, “Getting advice and signposting”, “Getting Help”, “Getting More Help” and “Getting Risk Support”. The model provides recommendations on how the experience of mental health services for the young person can be made less confusing and where shared decision-making with the children, adolescents and their families can take place. It lays stress on facilitating all the agencies involved in a child's or young person's life to work together in a focused and integrated way.

One of the main changes in the services – the components of transformation – is the introduction of a SPA (single point of access). This provides a single point of contact where children, young people and their parents can gain access to advice, consultation, assessment and treatment, where deemed appropriate, without the need for referral from a health care professional. Another change was the introduction of new service delivery pathways, whereby the young person's needs would be addressed within one integrated team, with additional speciality teams for eating disorders and neurodevelopmental conditions for example. Another component of transformation was the involvement of other parties to directly deliver particular services or to provide alternative or supplementary mental health support. The changes are in keeping with those taking place in other countries including Canada ( Malla *et al.* , 2019 ; Abba-Aji *et al.* , 2019 ), the US ( Cummings *et al.* , 2013 ), Australia and Ireland ( McGorry *et al.* , 2013 ).

There is a growing literature around the implementation of transformational change in health services. In a recent paper, Maniatopoulos *et al* . (2020) highlighted the importance of exploring and evaluating *“multiple levels of context”* which contribute to successful implementation of change, from policy to organisational responses to workforce settings, in order to see how they align or work against each other. However, to date, there has been little published evidence evaluating the changes undertaken in CAMHS. There is even less published on the perceptions and experiences of the mental health workforce around the CAMHS transformations. The studies that are published have highlighted two key, related areas. First, there have been longstanding concerns about staff retention in mental health services around the world ( Coates and Howe, 2015 ), and second, difficulties have been which identified when managing staff experiences within any type of service redesign ( Hung *et al.* , 2018 ).

In order to address the *“multiple levels of context”* which have a bearing on the success or otherwise of implementation of change, it is necessary to gain a ground-upwards view of what is happening and whether it is working. The rationale, therefore, for the study reported here, was that by gaining information from those who have been ultimately tasked with front-line delivery of the new services, we would be able to understand better the barriers and facilitators around implementation and how the changes can be maintained. The learning derived from such an exploration is the focus of this paper and its aim is to present findings on staff experience and perceptions of the changes in the transformation that their services underwent and implications of these for the workforce.

## Methods

### Study setting

Our study was conducted in South East England and included different CAMHS provided by Oxford Health NHS Foundation Trust, one of the largest CAMHS providers in England ( NHS Digital and Mental Health Services Monthly Statistics). The components of transformation adopted were a SPA with self-referral, new service delivery pathways, improved working with schools and partnership with third sector organisations in delivery of services.

### Design and research methods

We conducted a mixed methods observational study – the approach and methods are detailed in the study protocol ( Rocks *et al.* , 2018 ). Our main findings on the introduction of the SPA and on CAMHS outcomes are reported elsewhere ( Fazel *et al.* , 2021 ; Rocks *et al.* , 2020 ).

The qualitative study, in which an ethnographic approach was adopted to focus on the CAMHS transformations in Service C (peri-transformation) and Service E (post-transformation), is reported here. Between January 2018 and March 2019, field work was undertaken (by MS), which included 80 h of observation. This was mainly in Service C and included shadowing key staff; informal interviews with different stakeholders; as well as attending a range of team meetings **.** Observations were recorded in field notes and a field diary. They were coded and analysed thematically (by MS) and then critically discussed within the research team. The resulting themes fed into both the semi-structured interviews undertaken with staff and were re-read and incorporated into the overall thematic analysis presented in this paper.

In-depth, semi-structured interviews were also conducted with a range of staff members. In terms of sampling, we aimed to recruit and interview staff from diverse professional groups involved in different aspects of the transformation across the two services (C and E), in order to gain a range of experiences within each of the transformation components. Altogether 16 in-depth interviews took place, with an equal number across the two services. Staff members interviewed ranged from administrative and clinical staff, including those involved in the SPA, specialist services and third sector partners to service managers. The interviews were digitally recorded and professionally transcribed.

We drew on principles of grounded theory (GT) to guide our data collection and analysis ( Strauss and Corbin, 1998 ). GT is a set of systematic methods for carrying out qualitative research aimed at development of theory. It is however, possible to adopt principles of GT without aiming to generate theory ( Timonen *et al.* , 2018 ). This meant that we used early interviews to inform our approach to sampling and identifying further participants for interview, in order to capture as wide a range of experiences among the workforce as possible (theoretical sampling) ( Strauss and Corbin, 1998 ). The qualitative data were analysed thematically (by MS and MG). The analysis was guided by the constant comparative method ( Silverman, 2017 ), which included reading and familiarisation with the field notes and transcripts, noting initial themes and then conducting systematic and detailed open coding (adopted from GT) using NVivo 12, a qualitative data analysis software package which assists in the organisation and retrieval of data. The coding of the first set of interviews generated an initial coding framework or set of codes, which MS and MG discussed. This was further developed and refined as observation and analysis proceeded and developed further with the research team. The research team also critically discussed ideas for categories (provisional groupings of codes), and eventually the themes which emerged from the data, to ensure trustworthiness ( Lincoln and Guba, 1985 ).

The qualitative data reported in this paper represent two standpoints: the data from Service C were collected at the time of implementation capturing proximal changes (2018/19) and the Service E data were collected within a context of more distant change, two to three years post-transformation (2015/16). Awareness of the different contexts in which data were collected was sustained as analysis proceeded.

To ensure anonymity all quotations are labelled only according to the Service in which the staff member was located. Field notes often related to highly specific sessions such as staff meetings and for that reason, quotations from these notes have not been provided because of the possibility of identification of particular staff members.

## Findings

Analysis of the qualitative data revealed three main themes from the perspective of staff members. These were (1) the rationale for the transformation; (2) implementation of the transformation; and (3) maintenance of the transformation, describing the opportunities and challenges encountered as the process of change unfolded.

The themes and sub-themes are summarised and displayed in Table 1 .

1. Rationale for the transformation
  The overall transformation

Staff members were very supportive of the plan for transformation and what they believed it could achieve for children and young people (CYP):

My instinct was, it was the right model, with the right language with good principles and good thinking behind it and some evidence. [Service C]

Staff emphasised the principle of involving others to anticipate and prevent mental health issues in CYP and saw the transformation as a way of delivering this vision:

I think it's creative. I think it's much more kind of proactive … there is a lot more emphasis on trying to work in the community and the proactive kind of looking at the early signs to really try and support our colleagues … in more preventative type kind of measures. [Service E]

Components of the transformation

Among the components of the transformation, staff saw the SPA as the most significant of the changes, in the way it aimed to make the service accessible to all:

… anyone can access us and have a conversation as soon as they have got a question about mental health and we will listen to them and try and do something with that … For me, the biggest single change that we have managed to bring about lies in SPA. [Service C]

Staff regarded the SPA as a way of ensuring the right help was offered. While contact with SPA would not always lead to referral to the service, it would mean that callers would find out quickly:

it's just much more of a friendly front door to the service. But equally it does not mean that we're going to accept everybody. But if we're not going to accept you, we'll tell you, rather than you finding out in three weeks' time by a letter. [Service C]

Even if a referral was not made, the SPA team could still offer support and indicate resources which might be helpful:

it's not that we say ‘no' to anybody … There's always a signpost, or a recommendation, to every person that we speak to. I hope nobody goes away feeling ‘well that was pointless'. They might think ‘well I did not get the answer I wanted, but at least I know I can do this and this'. Which is much more helpful. [Service C]

The transformation in Service C saw a change in how CAMHS staff worked with secondary schools. In the previous service model, CAMHS workers had held a caseload in schools and work with CYP usually involved individual one-to-one contacts. The new model saw CAMHS “InReach” workers taking on a more advisory role, offering support and guidance to school staff and giving them tools to respond to mental health issues, such as feelings of anxiety, through training, workshops, assemblies and group work:

… the [school work] model … is all about empowering community, and staff, and building resilience. [Service C]

Staff viewed this change very positively and saw the importance of fostering school environments which were supportive of mental health, as a means of reducing the demand on CAMHS:

Schools InReach team are awesome …. the potential of what they can do has a far reaching impact. If you can get into schools as early as possible and we are hopefully doing ourselves out of a job. [Service C]

The configuration of CAMHS roles under the transformation also meant that staff did not have to automatically take on school work. CAMHS workers taking up the roles now chose to do so and felt more invested in the process:

that's really important, that the people here want to be here. Because that was not always the case, because we were just given a school before. Yeah. As part of the role. [Service C]

and staff believed that consequently schools would receive a better service:

the previous [school work] service did not work great for all schools. Because some people did not want to be in schools. Weren't confident to do training or assemblies, so did not offer that. So some schools were getting a really good service, others were not getting anything. [Service C]

Another component of the transformation saw the introduction of new partnerships with, and service delivery by, third sector organisations. In Service E, SPA was set up in partnership with Barnardo's, one of the largest charities providing care to children in England, in an attempt to improve the experience of support CYP and their families received. This represented a major change for the workforces of both CAMHS and Barnardo's. Barnardo's staff became Contact Support Workers (CSW) in SPA, where they took initial calls and then liaised with clinicians who were responsible for making decisions about referral.

In Service C, CAMHS aimed to work jointly with a range of organisations to provide alternative means for CYP to access help and support. Third sector organisations include art and creative based charities, woodwork workshops, food recycling and courses and training in employment matters and mechanics. Staff were positive about this aspect of the transformation because they saw it as another way in which CYP who might not have accessed the service previously could now do so:

… a lot of those charities naturally engage with a group that we have always struggled to engage with … not in education – that is really good. We have never managed to get them in mental health services before. Now we can see them through there, if they do not want a mental health record we can still see them. [Service C]

1. Implementation of the transformation
  Preparing for transformation

As they prepared for transformation, some staff members, particularly those in non-managerial roles, perceived a lack of communication and circulation of helpful information about the changes:

Personally, that's one of the biggest downfalls for me, the communication of it. Because I think everybody's heard kind of little bits. And depending which team meetings people have been to, you've heard less. I did go to the team meetings where they talked about transformation, but again it was very kind of woolly information. [Service C]

The service transformation required most staff to apply for their ongoing posts, determining which new roles they would take up and in some cases, they were competing with colleagues for those roles. This sometimes led to feelings of being undervalued, uncertainty and insecurity:

I think people could choose whether they went into SPA … and then if more than one person wanted the job they had to have a competitive interview … that obviously caused tension. [Service C]

At this time, with staff waiting to hear news about their new roles or contract renewal, other staff made the decision to leave the service altogether:

Things are tense … and some people are thinking of leaving, they're just waiting to hear. We've got lots of locums in. And so it's been quite, quite difficult. [Service C]

Staff members were aware of the importance of leadership for SPA at the time of its implementation. Some staff members emphasised the need for a manager responsible for each of the components of the transformation so that emerging difficulties would be recognised and managed:

There was not a manager before it started. And I have strong views that … if it's nobody's baby it will never, there is never anyone to … it never takes priority. [Service E]

Speed of implementation

When the transformation was implemented, change was felt to come about very quickly and there was a general feeling of unpreparedness:

I personally think that it was launched too early. When we went into that room, we did not have enough phones. The computers did not work. The white board was not even up and running. We had problems with connections to the phones. We did not have enough staff. [Service C]

Most staff felt that more time for training and planning would have helped to make a successful start to SPA:

we have not had any specific training. I think again, like most other things that have happened, it's been so quick, once it got started … there's then not that time for 'right, actually we're going to have a three day training programme on what the systems look like, what you're meant to be saying on the phone to these people'. [Service C]

especially as staff were taking on completely new roles within the service:

So people knee jerk and just start. Whereas actually it would be much better to say well, we are not going to start yet, because we need to put this in place and it will be successful … let's have a look at how referrals are going to come in. Who is going to triage them? Who's going to be trained to do that? Who's going to be trained in risk? [Service C]

Retaining and supporting staff

Challenges occurred early on in the change process around recruiting and retaining the right workforce. The work in SPA was a new direction for the service and some staff relished the opportunity to use their skills in a novel way:

initially I was a bit kind of worried that I was going to lose clinical skills … But actually, doing the telephones and things like that, I've actually probably used them more than I would in the one to one work. Because you're having to think on your feet so much quicker. [Service C]

However, others struggled, as this staff member recalled from the introduction of the SPA in Service E:

We have had a couple of staff that have not fitted. I think it is a particular role. [Service E]

The early days following the launch of SPA were extremely challenging for staff, when some were struggling with their new role, and this made early support vital. Other staff also mentioned the difficulties of working in new open plan settings where colleagues might overhear their phone discussions.

Establishing processes

As the transformation continued, staff found themselves dealing with issues for which they had had no chance to prepare, described as *“fire-fighting as we go along”.* Staff members quickly recognised the need for, and then proactively developed processes, which would guide them.

And we do have a script, which is helpful … what we could learn from it actually is to have just that time in between to go … “this is what you say on the phone to somebody”, “this is how you fill in one of the sheets and where it goes”. That sort of stuff would have been really, really helpful, I think. [Service C]

As well as being helpful to staff, this was also regarded as necessary to ensure consistency for those contacting SPA:

we need some kind of structure that we are all saying the same thing. A parent could call back in the next day and speak to somebody different and be told something completely different … that's a bit worrying … I know we have all got different backgrounds and we have all got different experiences. I think there should be some kind of structure to what we are saying to parents and professionals. [Service C]

One member of staff from Service E recalled that once a process had been set up, the work of SPA changed for the better:

[SPA] was doing a lot of the day to day stuff and then, but what we had not got then was any process. We had not got any stuff. But we had not got any process. And so the hardest bit was getting the process in. Once we got the process, the whole world changed.” [Service E]

Managing new relationships

In Service E, the setting up of SPA in partnership with a third sector organisation required the management of new relationships and represented a challenge for both sets of staff, from Barnardo's as well as the NHS:

the call handlers were Barnardo's staff. They had just come into mental health and into the health service. So for them it was working under the NHS … so the process, the governance, the previous experiences would be very different … it was a big leap into this massive organisation that has processes for everything, literally everything. And I guess from NHS staff point of view, there was probably some anxiety about how do we work with a third party. We have not done it before … how do we support them in getting to know the NHS … [Service E]

Staff recalled that the SPA staff, with different backgrounds and coming from different organisational “cultures” needed to find a way to work together very quickly:

I guess to build those relationships and for them to be introduced to kind of the management of CAMHS and it was yeah, having a new relationship. It was having a new best friend that you had to develop the relationship really quickly. [Service E]

The need for support for the Barnardo's call handlers was also underlined, as they were exposed to potentially upsetting situations in the phone conversations they were having and to ensure that they were working within boundaries:

It's making sure that the call handlers are supported as well, because they are very likely to come across any kind of distressing or difficult situations. And also just ensuring that they are not giving kind of clinical advice when they are not kind of in a position to be able to do that. [Service E]

1. Maintenance of the transformation
  Staffing as a key issue

Staff in Service E, from their standpoint of more distant change, highlighted the importance of having adequate levels of consistent staffing as a starting point, to ensure that transformation could proceed smoothly and be maintained:

going into a new model with limited permanent clinical staff was hard. So there was a lot of locum staff and I think that made it difficult in the sense of the consistency and the culture change. [Service E]

Staff recognised the success of SPA in terms of increasing accessibility:

It shows actually that the service is working, because people are feeling confident to refer. I think that's why it's increased … people have a better understanding of what is there. [Service E]

However, staff perceived that demand for CAMHS was rising and was likely to continue rising. Having enough staff to cope with this demand was seen as key:

In terms of the SPA team, think we will continue to grow, because the demands on the service will keep growing. But that needs to be met by increasing staff. [Service C]

In the longer term, staff questioned the sustainability of this aspect of the transformation unless staff numbers could be increased:

So, it has been noted that actually the majority of people in SPA are doing over their working hours because there are not enough hours in the day for the capacity of work that we're doing. [Service C]

Integrating teams

Staff reflected that the lack of contact and shared space between NHS and partner organisation staff was a barrier to change. This was recognised as an essential part of maintaining and moving the transformation on:

I think reflecting back, we were in our room by ourselves, but actually, if we had been mixed and put in with everybody else, that kind of transition and that getting to know you would have happened a lot quicker. I definitely think that's key, because it's being part of this big team and it's not being a partner. [Service E]

Establishing close working has been addressed but staff perceived that it should not have taken as long as it did:

it's definitely more of a collaborative way of working. I think the relationships are sufficiently strong that we can pick up the phone and say, actually, can we just talk about it … it's taken longer than I would have anticipated. Perhaps I have just underestimated. [Service E]

Likewise, staff perceived that relationships between the different components of the service could impact negatively on the ongoing transformation and that the teams needed to work together:

one of the cons of pathways is it can sometimes feel like mini teams with their own agendas. Everyone has their own pressures … sometimes it feels like we are all working in isolation. Just because you solve your problem, just make sure that the problem has been solved and not just moved … so it's about making sure that we are on the same team. [Service E]

Sharing experiences

Staff in Service E CAMHS felt that the transformation process had meant being able to work to build resilience in the community and in a more evidence-based way:

I think there is a lot more emphasis on trying to work in the community … in kind of more preventative type kind of measures … So there seems to be a bit more about doing things differently, but based on the right standards and the right clinical guidance. There is more of a link I guess also to evidence and research, rather than just, we've always done it this way. [Service E]

The resolve of the workforce in implementing and maintaining the transformation was also present in the accounts of staff:

I think the resilience of everybody, actually, Service E and Barnardo's going along to lots of different meetings. Being part of their team meetings … So that helped that transition … it did take time … I do not feel like them and us. I feel like we are part, we are all together. [Service E]

Staff members in Service E, aware of how much they had learnt through the process of transformation, welcomed the opportunity to share their experiences with other services where transformation was planned:

Because so much of it was working it out as we went along. I do not even know if anything – how can you work something out if you do not know at least who worked out until you are there. [Service E]

## Discussion

Our main findings show that staff members were supportive of the transformation and the underlying principle of increasing accessibility. Their accounts reveal that the transformation process had important impacts on the workforce at the time, there was a necessity to recruit more staff to ensure access rates for children and adolescents could be maintained. The earliest stages of the transformation were perceived by front-line staff as lacking sufficient communication and planning, resulting in unpreparedness which was de-stabilising. Staff would have welcomed more time for planning and training. In a similar way, staff embraced the new ways of working with schools and partner organisations. However, the tight time-frames to set up processes with, and manage expectations of other organisations, meant that change did not always start smoothly. Time spent on planning could have mitigated some of the issues.

Overall there is a small amount, although growing, of research evidence specifically on the CAMHS transformations taking place world-wide. However, we are aware of very few which have set out to fully investigate the experiences and perceptions of CAMHS staff journeying through transformation. Our focus on a particular context (staff views and experiences) and setting where transformation was taking place therefore represents an original contribution. In addition, our study presents data around CAMHs transformations from two standpoints. This combination is unusual and offers particular insights. It is notable that a number of issues experienced and reflected on by Service E (namely, finding and retaining the right staff for the SPA whose workload was increasing; the need to quickly put in place processes for handling calls; and building relationships with other agencies) were being contemporaneously experienced by Service C. While changes in CAMHS are occurring across the UK, the same changes are not occurring in all services at the same time. Thus, sharing of learning around good practice in implementing changes by one service with another embarking on the same changes could be possible and useful. Indeed, in the case of Services C and E operating within the same trust, the opportunity for more “local” communities of learning would have been a feasible proposition.

While there are no similar studies describing the experiences and perceptions of a mental health workforce undergoing transformation elsewhere, our findings resonate with the broader literature around changes in mental health services and the NHS in general. The staff members we interviewed were positive about the changes being proposed in CAMHS, although they did not feel they had a chance to provide input into them and often lacked full knowledge of their practical service implications. The concerns voiced by staff reflect the top-down nature of the transformation and the uncertainty it generated. Jun *et al* . (2014) described this approach to service re-design that is led by government policies, as often focussing on location-specific implementation rather than the generation of new ideas for service design and thus creating barriers to successful implementation. For the staff in our study, both those who had already experienced transformation and those in the midst of change, a lack of awareness and understanding of the proposed changes was a notable early barrier.

Staff saw themselves and their roles as key in delivering the transformed services and outlined the different pressures they faced in the context of the changes. In their qualitative exploration of stakeholder perceptions of quality care, Svirydzenka *et al* . (2017) concluded that quality care ultimately relied on staff feeling *“supported, valued, rested and motivated”* . Such needs were also prominent in the accounts of the staff included in our study. In their evaluation of more general transformational change in the NHS, Hunter *et al* . (2015) emphasised the time it can take for change to become embedded, especially when the change is a cultural one. They argued that managing to retain their stability at the time of change is problematic for organisations, as external pressures are likely to intrude. Our findings reflect this as the changes were externally driven and so the time-frames were imposed on the services leading to perceptions in the workforce of a lack of stability as they underwent transformation. This then made the task of generating consistent structures and processes within the new services considerably more difficult.

The COVID-19 global pandemic has, arguably, made it even more important to understand the perspectives of front-line staff. CAMHS services have likely had to adapt again at a time when they are already attempting to maintain the transformations, meaning the impact on staff is likely to be even greater. Further research is needed to understand the facilitators and barriers to maintaining transformational change, particularly in the light of the pandemic. A further research priority is the generation of evidence from the perspectives of the children, young people and their families who access the transformed CAMHS.

## Strengths and limitations

We believe that the “triangulation” achieved through the collection of observation and interview data around transformation provides a more complete picture than interviews alone. We were also able to gather data from a range of different staff involved in the transformation and from two linked CAMHS at different stages of the transformation. We also believe that the descriptions of context and the components of transformation undergone will enable readers to assess how our findings might relate to and apply in their own context. This is the concept of “transferability” ( Lincoln and Guba, 1985 ), whereby researchers provide thorough information about context and how the data were collected, in order to enable others to “transfer” the results to their own situation.

Further follow-up of the service undergoing transformation would have provided longitudinal data and useful information about the ongoing process of change ( Pandhi *et al.* , 2018 ).

## Conclusions

This paper presents evidence around the perceptions and concerns of a mental health workforce, whose organisation has undergone significant service reconfiguration and change. Staff concurred with the need for service design changes and demonstrated considerable positivity and resilience in the face of change. However, in taking better account of their needs, staff retention and satisfaction might have been improved. At early stages of “transformation”, it is important to find ways to make time for and encourage staff to provide ideas and input into the components of change that they can influence, as well as find a variety of ways to keep them informed. In this study, although senior staff managed all aspects of the service changes which ensured that the change took place in as uniform manner as possible across the different components of services, this approach could also introduce new difficulties or inequalities. Allowing staff time for planning and training might enable them to better develop the processes and resources they will require and is crucial to any implementation plan of service transformation. Developing ways for services to compare changes they are planning to implement and then sharing good practice around implementation with each other is also vital.

## Figures and Tables

**Table 1:** Summary of themes and sub-themes

Theme	Sub-themes
Rationale for the transformation	*The overall transformation*
	*Components of the transformation*
Implementation of the transformation	*Preparing for transformation*
	*Speed of implementation*
	*Retaining and supporting staff*
	*Establishing processes*
	*Managing new relationships*
Maintenance of the transformation	*Staffing as a key issue*
	*Integrating teams*
	*Sharing experiences*
